# Shock Index-C: An Updated and Simple Risk-Stratifying Tool in ST-Segment Elevation Myocardial Infarction

**DOI:** 10.3389/fcvm.2021.657817

**Published:** 2021-06-15

**Authors:** Peng Ran, Xue-biao Wei, Ying-wen Lin, Guang Li, Jie-leng Huang, Xu-yu He, Jun-qing Yang, Dan-qing Yu, Ji-yan Chen

**Affiliations:** ^1^Guangdong Provincial Key Laboratory of Coronary Heart Disease Prevention, Department of Cardiology, Guangdong Cardiovascular Institute, Guangdong Provincial People's Hospital, Guangdong Academy of Medical Sciences, Guangzhou, China; ^2^Department of Critical Care Medicine, Guangdong Provincial Geriatrics Institute, Guangdong Provincial People's Hospital, Guangdong Academy of Medical Sciences, Guangzhou, China

**Keywords:** shock index, renal function, ST-segment elevation myocardial infarction, percutaneous coronary intervention, major adverse clinical events

## Abstract

**Background:** Shock index (heart rate/systolic blood pressure, SI) is a simple scale with prognostic value in patients with ST-segment elevation myocardial infarction (STEMI) undergoing percutaneous coronary intervention (PCI). The present study introduces an updated version of SI that includes renal function.

**Methods:** A total of 1,851 consecutive patients with STEMI undergoing PCI were retrospectively included at Cardiac Care Unit in Guangdong Provincial People's Hospital and divided into two groups according to their admission time: derivation database (from January 2010 to December 2013, *n* = 1,145) and validation database (from January 2014 to April 2016, *n* = 706). Shock Index-C (SIC) was calculated as (SI × 100)–estimated CCr. Calibration was evaluated using the Hosmer-Lemeshow statistic. The predictive power of SIC was evaluated using receiver operating characteristic (ROC) curve analysis.

**Results:** The predictive value and calibration of SIC for in-hospital death was excellent in derivation [area under the curve (AUC) = 0.877, *p* < 0.001; Hosmer-Lemeshow chi-square = 3.95, *p* = 0.861] and validation cohort (AUC = 0.868, *p* < 0.001; Hosmer-Lemeshow chi-square = 5.01, *p* = 0.756). SIC exhibited better predictive power for in-hospital events than SI (AUC: 0.874 vs. 0.759 for death; 0.837 vs. 0.651 for major adverse clinical events [MACEs]; 0.707 vs. 0.577 for contrast-induced acute kidney injury [CI-AKI]; and 0.732 vs. 0.590 for bleeding, all *p* < 0.001). Cumulative 1-year mortality was significantly higher in the upper SIC tertile (log-rank = 131.89, *p* < 0.001).

**Conclusion:** SIC was an effective predictor of poor prognosis and may have potential as a novel and simple risk stratification tool for patients with STEMI undergoing PCI.

## Introduction

Despite advanced evidence-based medical treatments and the widespread use of percutaneous coronary intervention (PCI), ST-segment elevation myocardial infarction (STEMI) is a leading cause of mortality worldwide (1). The in-hospital mortality rate for STEMI is 3–4% following PCI, and can reach up to 10% in any given year (2, 3). In addition, increased risk of bleeding and acute kidney injury was observed in patients with STEMI, which was associated with poor outcomes (4, 5). Many scales have been developed for early identification of patients with STEMI at high risk for adverse outcomes. Global Registry of Acute Coronary Events (GRACE) and Thrombolysis in Myocardial Infarction (TIMI) risk scales are guideline-recommended risk stratification tools for prediction of STEMI-related mortality (6). Mehran et al. (7) and Can Rapid Risk Stratification of Unstable Angina Patients Suppress Adverse Outcomes With Early Implementation of the American College of Cardiology/American Heart Association Guidelines (CRUSADE) scores are used to predict contrast-induced acute kidney injury (CI-AKI) and bleeding, respectively (8). Each score predicts a different event, and collection of data and calculation of these four scores is time consuming in clinical practice. A simpler scoring system that can simultaneously assess the risk of mortality, bleeding, CI-AKI, and other STEMI-related adverse events is of critical importance.

Shock index (SI), defined as the ratio of heart rate to systolic blood pressure, is a simple risk-stratification tool used to evaluate patients with STEMI (9). However, the discriminatory ability of SI for short- and long-term adverse events is considered insufficient (10). In addition to heart rate and systolic blood pressure, renal function is an essential element of the GRACE, Mehran and CRUSADE scales (7, 11, 12), but the effect of addition of renal function to SI on prediction of poor prognosis has not been characterized. In this study, we developed a new model, Shock Index-C [SIC; (SI × 100)-estimated creatinine clearance rate (CCr)], and validated its predictive ability in patients with STEMI undergoing PCI.

## Methods

### Patients and Study Design

We retrospectively enrolled 1,907 consecutive patients with acute STEMI undergoing PCI at the Cardiac Care Unit in Guangdong Provincial People's Hospital between January 2010 and April 2016. STEMI was diagnosed according to the American College of Cardiology Foundation/American Heart Association guidelines (6). The exclusion criteria were as follows: (1) Hospital stay <24 h; (2) malignant tumor; (3) concomitant aortic dissection; and (4) missing admission serum creatinine (Scr) data. After screening, 1,851 patients were included (Supplementary Figure 1). This study was approved by the Ethics Committee of our hospital, and the requirement for informed consent was waived due to the retrospective nature of the study.

### Clinical Data and SIC

Clinical data were collected from the electronic case report form by one researcher and randomly confirmed by another researcher. Data collected included patient demographics, previous medical history, laboratory results, PCI procedural details, adverse events, and medical treatment. Vital signs including blood pressure and heart rate were obtained from data recorded at admission. SI was calculated using the following formula: heart rate (bpm)/systolic blood pressure (mmHg). Estimated creatinine clearance rate (CCr) was calculated with the published equations for Cockcroft-Gault: man: (140–age)/ Scr, woman:(140–age) /Scr × 0.85 (13). SIC was calculated using the following formula: (SI × 100)–estimated CCr. GRACE, CRUSADE, Mehran, and TIMI risk scores were calculated using initial clinical history, electrocardiograms, laboratory values, and PCI procedural information collected at admission.

### Follow-Up and Endpoints

All surviving in-hospital patients were followed-up through telephone interviews. We reviewed hospital readmission records and outpatient clinic interviews for possible events. The primary endpoints were in-hospital and 1-year mortality. In-hospital major adverse clinical events (MACEs) such as stroke, dialysis, acute heart failure, and target vessel revascularization (TVR) during hospitalization were used as a composite end point. In addition, CI-AKI and bleeding were recorded. Contrast-induced acute kidney injury was defined as elevation of Scr by 50%, or 0.3 mg/dL from baseline, within 48 h, according to the Kidney Disease: Improving Global Outcomes criteria (14).

### Statistical Analysis

Normally distributed continuous data are presented as the mean ± standard deviation, and non-normally distributed continuous data are presented as the median and interquartile range, which were compared using independent sample *t* and non-parametric tests, respectively. Categorical variables are displayed as numbers and percentages, were compared using chi-square tests. The Hosmer-Lemeshow goodness-of-fit test was used to assess the goodness of fit of the final regression model. Receiver operating characteristic (ROC) curve analysis was conducted to determine the optimal cut-off levels of SIC for prediction of adverse events. Decision curve analysis was used to compare the predicting performance for in-hospital mortality by quantifying the net benefits. Area under the curve values were compared using the nonparametric approach described by DeLong et al. (15). In addition, net reclassification improvement (NRI) and integrated discrimination improvement (IDI) were calculated. The performance of SIC in subgroups was further assessed using ROC curves. Variables significantly associated with mortality in the univariate analysis were included in the multivariable analysis. Kaplan-Meier curves were generated and compared using the log-rank test. *P* < 0.05 were considered statistically significant. All analyses were conducted using SPSS software (version 22.0; SPSS Inc, Chicago, IL, USA).

## Results

### Patient Characteristics

A total of 1,851 patients with STEMI undergoing PCI were included. Baseline characteristics are shown in Table 1. The mean age was 61.4 ± 12.3 years and 1,532 (82.8%) of the included patients were male. They were divided into two groups according to their admission time: derivation database (from January 2010 to December 2013, *n* = 1,145) and validation database (from January 2014 to April 2016, *n* = 706). Higher SI, lower CCr and left ventricular ejection fraction (LVEF), less stents implantation and usage of Angiotensin-Converting Enzyme Inhibitors (ACEIs) /Angiotensin Receptor Blockers (ARBs) and β-blocker were found in validation database (Table 1). Except for that, there were no significant differences in the clinical features between two groups.

**Table 1:** Baseline characteristics of patients in the model derivation and validation database.

**Variables**	**All** ** (*n* = 1,851)**	**Derivation** ** (*n* = 1,145)**	**Validation** ** (*n* = 706)**	***P*-value**
**Age, years**	61.4 ± 12.3	61.2 ± 12.2	61.9 ± 12.4	0.236
Age 65–74 years	480 (25.9)	307 (26.8)	173 (24.5)	0.103
Age ≥75 years	297 (16.0)	168 (14.7)	129 (18.3)	
**Gender**, ***n*** **(%)**				0.932
Male	1,532 (82.8)	947 (82.7)	585 (82.9)	
Female	319 (17.2)	198 (17.3)	121 (17.1)	
**Medical history**, ***n*** **(%)**				
Smoke	834 (45.1)	502 (43.8)	332 (47.0)	0.181
Diabetes	459 (24.8)	281 (24.5)	178 (25.2)	0.745
Hypertension	941 (50.8)	591 (51.6)	350 (49.6)	0.394
PVD	30 (1.6)	21 (1.8)	9 (1.3)	0.355
MI	89 (4.8)	60 (5.2)	29 (4.1)	0.269
PCI	179 (9.7)	116 (10.1)	63 (8.9)	0.393
Weight, kg	65.3 ± 10.9	65.2 ± 10.7	65.6 ± 11.1	0.405
Cardiac arrest before admission, *n* (%)	45 (2.4)	26 (2.3)	19 (2.7)	0.568
Time to admission ≤ 24 h	1,367 (73.9)	834 (72.8)	533 (75.5)	0.206
**Blood pressure, mmHg**				
Systolic	121.4 ± 22.0	122.1 ± 22.5	120.2 ± 21.1	0.071
Diastolic	73.3 ± 13.1	73.3 ± 13.1	73.4 ± 13.3	0.941
Heart rate, bpm	80.2 ± 16.1	79.8 ± 16.0	80.8 ± 16.2	0.181
Shock index × 100	68.2 ± 19.6	67.5 ± 19.5	69.3 ± 19.6	0.048
Killip ≥ 2	529 (28.6)	324 (28.3)	205 (29.0)	0.732
Serum creatinine, mg/dL	1.0 (0.8, 1.2)	1.0 (0.8, 1.2)	1.0 (0.8, 1.2)	0.188
CCr, ml/min	80.8 ± 30.5	82.0 ± 31.1	79.0 ± 29.3	0.039
Hemoglobin, g/L	132.4 ± 17.1	132.3 ± 16.7	132.6 ± 17.7	0.654
LVEF, %	52.2 ± 11.1	52.7 ± 10.6	51.4 ± 11.7	0.019
IABP, *n* (%)	202 (10.9)	116 (10.1)	86 (12.2)	0.169
**Culprit vessel**				0.718
Left main	63 (3.4)	40 (3.5)	23 (3.3)	
LAD	957 (51.7)	591 (51.6)	366 (51.8)	
LCX	191 (10.3)	122 (10.7)	69 (9.8)	
RCA	639 (34.5)	392 (34.2)	247 (35.0)	
SVGs	1 (0.1)	0 (0)	1 (0.1)	
**Treated vessel**, ***n*** **(%)**				
Left main	74 (4.0)	46 (4.0)	28 (4.0)	0.956
LAD	1,083 (58.5)	671 (58.6)	412 (58.4)	0.917
LCX	303 (16.4)	185 (16.2)	118 (16.7)	0.753
RCA	704 (38.0)	433 (37.8)	271 (38.5)	0.807
Multi-vessel	232 (12.5)	139 (12.1)	93 (13.2)	0.514
Number of stents	1.4 ± 0.8	1.4 ± 0.8	1.3 ± 0.7	0.040
**In-hospital medication**, ***n*** **(%)**				
DAPT	1,819 (98.3)	1,129 (98.6)	690 (97.7)	0.164
Aspirin	1,831 (99.0)	1,136 (99.2)	695 (98.6)	0.190
Clopidogrel	1,835 (99.1)	1,138 (99.4)	697 (98.7)	0.134
ACEs or ARBs	1,587 (85.7)	1,049 (91.6)	538 (76.2)	<0.001
β-blocker	1,578 (85.3)	1,018 (88.9)	560 (79.3)	<0.001
Statin	1,841 (99.5)	1,142 (99.7)	699 (99.0)	0.080
Hospital stay (days)	7 (5.9)	7 (5.9)	7 (6.9)	0.066

*ACEIs, Angiotensin-Converting Enzyme Inhibitors; ARBs, Angiotensin Receptor Blockers; CCr, creatinine clearance rate; DAPT, Dual Anti-Platelet Therapy; IABP, intra-aortic balloon pump; LVEF, left ventricular ejection fraction; PVD, peripheral vascular diseases; SVGs, saphenous vein grafts*.

During hospitalization, 68 patients (3.7%) died, 118 (6.4%) suffered acute heart failure, 70 (3.8%) received hemodialysis treatment, 12 (0.6%) received TVR, and 23 (1.2%) had strokes. The rate of CI-AKI and any bleeding were 10.0% and 10.4%, respectively. There was no statistical difference for in-hospital events between derivation and validation database, except for dialysis (3.0% vs. 5.1%, *p* = 0.02; Table 2).

**Table 2:** In-hospital outcomes in the model derivation and validation database.

**Variables**	**All** ** (*n* = 1,851)**	**Derivation** ** (*n* = 1,145)**	**Validation** ** (*n* = 706)**	***P*-value**
Stroke	23 (1.2)	13 (1.1)	10 (1.4)	0.596
Acute heart failure	118 (6.4)	68 (5.9)	50 (7.1)	0.328
TVR	12 (0.6)	6 (0.5)	6 (0.8)	0.582
Dialysis	70 (3.8)	34 (3.0)	36 (5.1)	0.020
Death	68 (3.7)	40 (3.5)	28 (4.0)	0.600
MACEs	178 (9.6)	101 (8.8)	77 (10.9)	0.139
CI-AKI	185 (10.0)	115 (10.0)	70 (9.9)	0.929
Bleeding	193 (10.4)	126 (11.0)	67 (9.5)	0.300

*CI-AKI, contrast-induced acute kidney injury; MACEs, major adverse clinical events; TVR, target vessel revascularization*.

### Development and Validation of SIC

**Figure 1:**
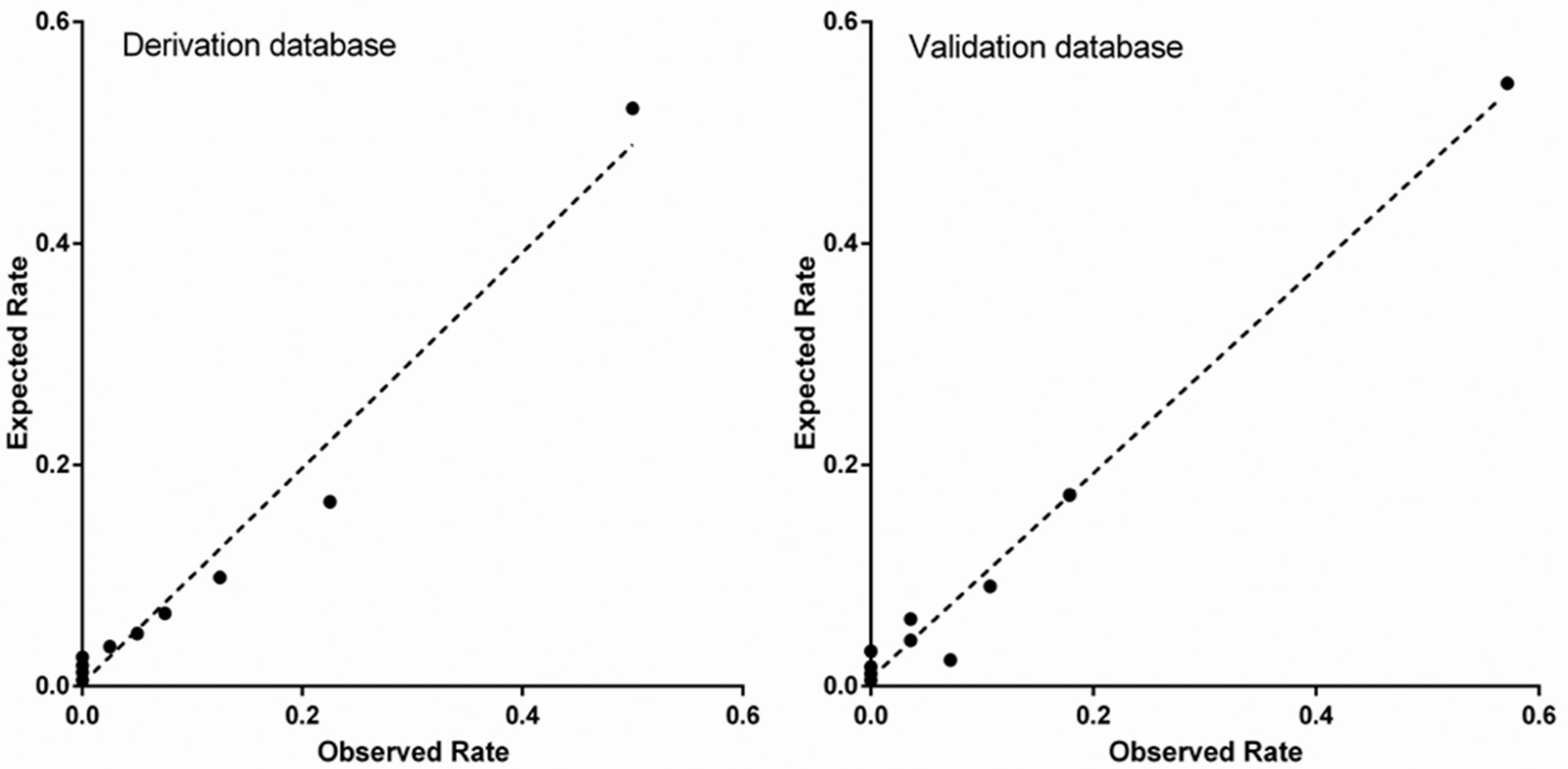
Calibration of shock index-C for in-hospital mortality in the derivation and validation database.

**Figure 2:**
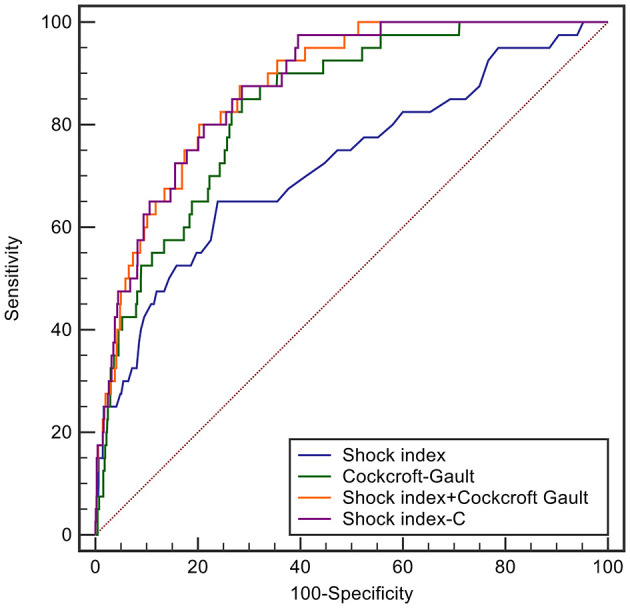
ROC curves of shock index, Cockcroft-Gault, shock index-C, and the prediction score developed by including the shock index and Cockcroft-Gault in the logistic model for in-hospital mortality in the derivation database.

**Figure 3:**
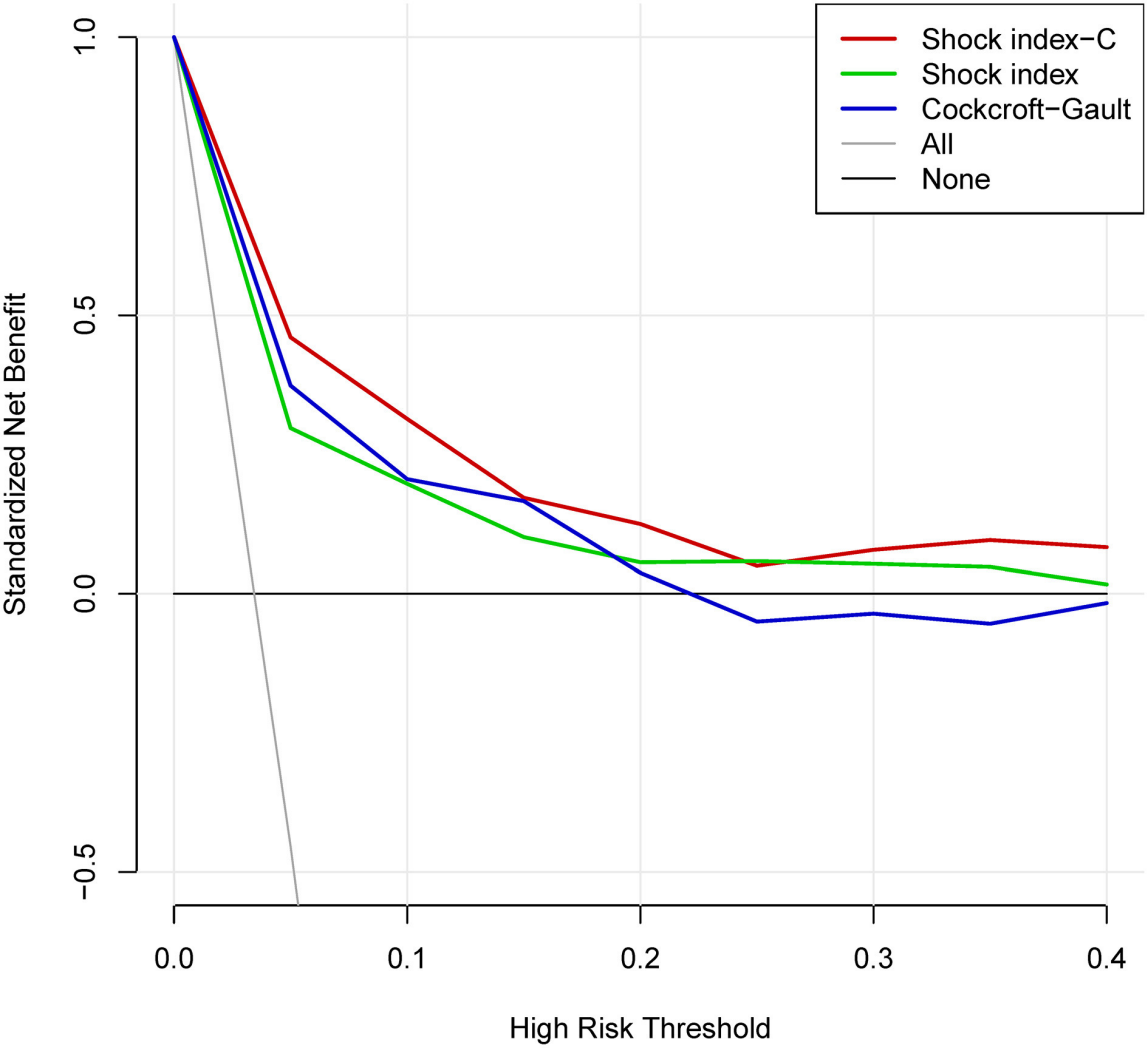
Decision curve analysis of shock index-C and shock index for in-hospital death in the derivation database.

In derivation database, the significant factors except the elements of SI and CCr in the univariate logistic regression analysis for in-hospital mortality were included into multivariate model (Supplementary Table 1). Cardiac arrest before admission, CCr, LVEF, and IABP were risk factors for in-hospital death independently of SI. Renal function is an essential element of previous scores in STEMI patients. Therefore, we added CCr into SI to create a new variable, SIC. The chi-square statistic for calibration was 3.95 (*p* = 0.861, Figure 1), indicating good discriminatory power and goodness-of-fit. Receiver operating characteristics curve analysis was performed to determine the predictive power of SI, CCr, the combination of SI and CCr using a logistic model (linear combination predictor), and SIC, for in-hospital death in derivation database. SIC had an excellent predictive value for in-hospital mortality (AUC = 0.877, 95% CI: 0.833–0.921, *p* < 0.001). In addition, the predictive power was higher than SI (AUC: 0.877 vs. 0.723, *p* < 0.001; NRI = 37.0%, 95% CI: 17.4–56.6, *p* < 0.001; IDI = 7.5%, 95% CI: 4.2–10.8, *p* < 0.001; Figure 2) and relatively higher than CCr (AUC: 0.877 vs. 0.838, *p* = 0.058; NRI = 21.1%, 95% CI: −3.6–45.7, *p* = 0.094; IDI = 6.6%, 95% CI: 0.5–12.8, *p* = 0.034; Figure 2). The decision curves analysis showed that SIC had the highest overall net benefit compared with SI and CCr (Figure 3). The AUC values for SIC and the linear combination predictor were similar (AUC: 0.877 vs. 0.876, *p* = 0.781, Figure 2). SIC was selected due to ease of clinical use. The AUC of SIC for in-hospital mortality was 0.868 (95% CI: 0.803–0.934) and was calibrated with a Hosmer-Lemeshow chi-square statistic of 5.01 (*p* = 0.756, Figure 1) in validation database.

### Subgroup Analysis

In addition, the SIC also exhibited good discrimination among subgroups in the overall population (Table 3), such as age, gender, and infarcted area.

**Table 3:** Subgroup analysis.

**Subgroup**	**Patients,** ***n***	**Deaths,** ***n***	**Hosmer–Lemeshow** ***P*-value**	**AUC (95%CI)**
**Age**				
<60	797	13	0.933	0.918 (0.843, 0.992)
≥60	1,054	55	0.713	0.825 (0.774, 0.876)
**Gender**				
Female	319	17	0.927	0.916 (0.866, 0.966)
Male	1,532	51	0.939	0.861 (0.815, 0.907)
**Infarcted area**				
Anterior	851	35	0.452	0.912 (0.881, 0.943)
Non-anterior	1,000	33	0.627	0.834 (0.770, 0.897)

### Comparison of SIC With Previous Risk Scores

**Figure 4:**
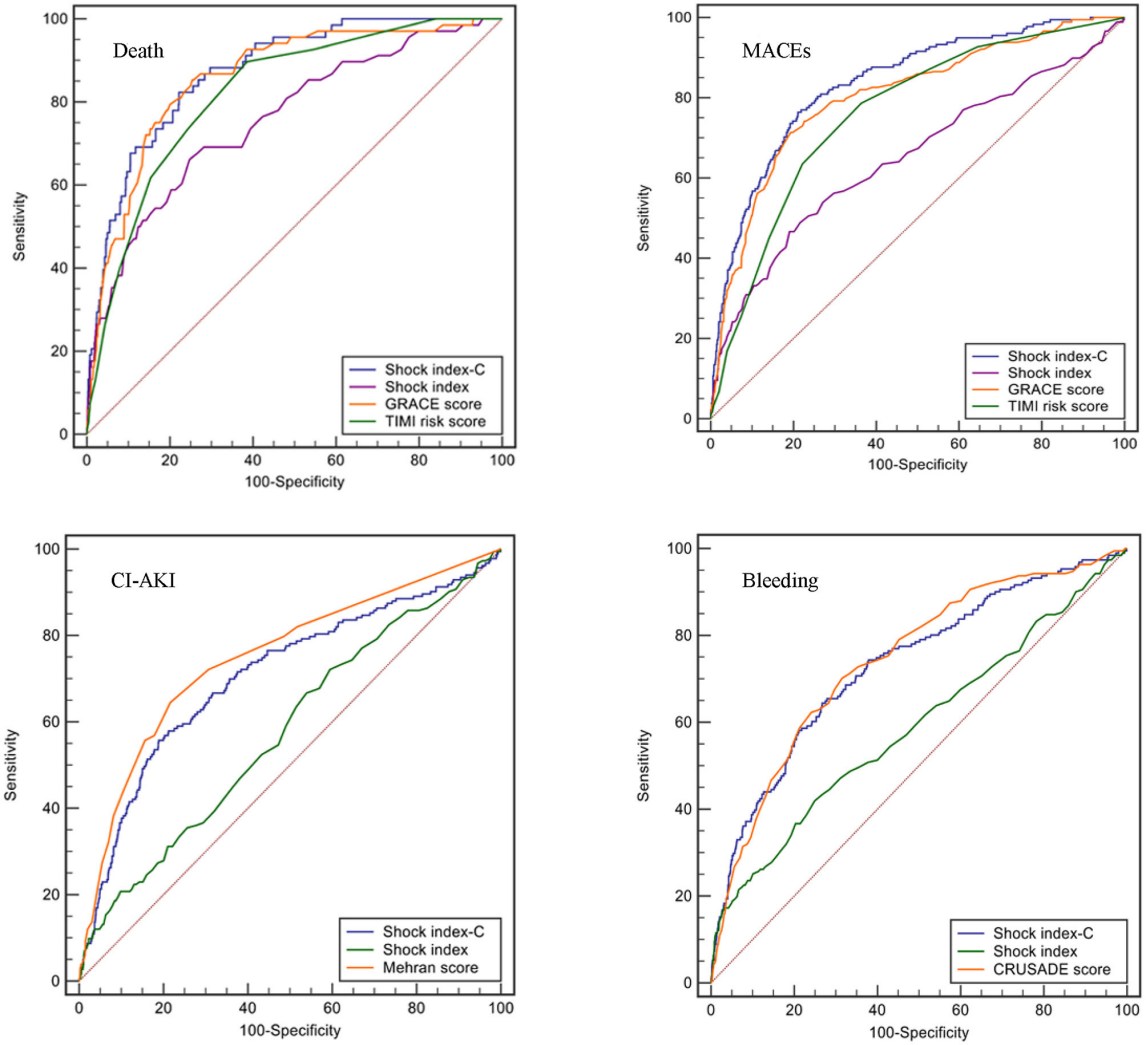
ROC curves of different scores for in-hospital events (death, MACEs, CI-AKI, and bleeding) in the overall population.

The discriminatory ability of SIC for in-hospital death, MACEs, CI-AKI, and bleeding was compared with other commonly used scales in the overall population. SIC exhibited similar predictive discrimination as GRACE (AUC: 0.874 vs. 0.859, *p* = 0.453; NRI = 16.5%, 95% CI: −2.1–35.1, *p* = 0.082; IDI = 3.6%, 95% CI: −0.3–7.6, *p* = 0.072; Figure 4; Supplementary Table 2) and higher than TIMI risk score (AUC: 0.874 vs. 0.822, *p* = 0.006, NRI = 20.1%, 95% CI: 0.7–39.5, *p* = 0.042; IDI = 7.5%, 95% CI: 2.9–12.1, *p* = 0.001; Figure 4; Supplementary Table 2) for in-hospital death. SIC was a better predictor of in-hospital MACEs than GRACE (AUC: 0.837 vs. 0.804, *p* = 0.008, NRI = 17.4%, 95% CI: 5.0–29.8, *p* = 0.006; IDI=4.4%, 95% CI: 1.6–7.3, *p* = 0.002; Figure 4; Supplementary Table 2) and TIMI risk score (AUC: 0.837 vs. 0.762, *p* < 0.001, NRI = 53.6%, 95% CI: 40.7–66.4, *p* < 0.001; IDI = 11.1%, 95% CI: 8.2–13.9, *p* < 0.001; Figure 4; Supplementary Table 2). For predicting CI-AKI, SIC did not perform well-compared with Mehran score (AUC: 0.707 vs. 0.749, *p* = 0.029; NRI = −41.5%, 95% CI: −54.8 to −28.2, *p* < 0.001; IDI = −4.2%, 95% CI: −6.0 to −2.4, *p* < 0.001; Figure 4; Supplementary Table 2). No difference was observed for prediction of in-hospital bleeding between SIC and CRUSADE score (AUC: 0.732 vs. 0.743, *p* = 0.380; NRI = −8.7%, 95% CI: −20.8–3.5, *p* = 0.161; IDI = 0.3%, 95% CI: −1.3–1.9, *p* = 0.691; Figure 4; Supplementary Table 2). In addition, SIC was a better predictor than SI for in-hospital death (AUC: 0.874 vs. 0.759, *p* < 0.001, Figure 4), MACEs (AUC: 0.837 vs. 0.651, *p* < 0.001, Figure 4), CI-AKI (AUC: 0.707 vs. 0.577, *p* < 0.001, Figure 4), and bleeding (AUC: 0.732 vs. 0.590, *p* < 0.001, Figure 4).

### Clinical Application of SIC

We categorized SIC into tertiles as follows to enhance its clinical utility: < -30 (*n* = 584), −30 to −5 (*n* = 567), and ≥-5 (*n* = 700). The incidences of in-hospital mortality (0 vs. 1.4 vs. 8.6%, *p* < 0.001, Supplementary Figure 2), MACEs (1.5 vs. 3.7 vs. 21.1%, *p* < 0.001, Supplementary Figure 2), CI-AKI (5.0 vs. 5.5 vs. 17.9%, *p* < 0.001, Supplementary Figure 2), and bleeding (3.8 vs. 6.9 vs. 18.9%, *p* < 0.001, Supplementary Figure 2) were significantly higher in patients in the upper SIC tertile. The cumulative 1-year mortality risk was significantly higher in the third tertile (log-rank = 131.89, *p* < 0.001, Supplementary Figure 3). The second (adjusted HR = 2.86, 95% CI: 1.06–7.74, *p* = 0.038, Supplementary Table 3) and third SIC tertile (adjusted HR = 7.80, 95% CI: 3.08–19.79, *p* < 0.001, Supplementary Table 3), compared to the first SIC tertile, was an independent risk factor for 1-year mortality.

The included patients were divided into two groups using an SIC cutoff of 10 (sensitivity 82.4%; specificity 77.8%) based on the ROC curve for in-hospital death: >10 (*n* = 450) and ≤ 10 (*n* = 1,401). The incidences of in-hospital mortality (1.0 vs. 12.0%, *p* < 0.001, Supplementary Figure 2), MACEs (3.5 vs. 28.7%, *p* < 0.001, Supplementary Figure 2), CI-AKI (5.7 vs. 23.3%, *p* < 0.001, Supplementary Figure 2), and bleeding (6.0 vs. 24.2%, *p* < 0.001, Supplementary Figure 2) were significantly higher in patients with SIC > 10. Patients with SIC > 10 were at higher risk for one-year mortality than those with SIC ≤ 10 (log-rank = 176.25, *P* < 0.001, Supplementary Figure 3). Multivariate Cox survival analysis indicated that SIC > 10 was independently associated with one-year mortality (adjusted HR = 3.13, 95% CI: 2.01–4.87, *p* < 0.001, Supplementary Table 3).

## Discussion

This study was the first to combine SI and renal function for determination of prognosis of patients with STEMI undergoing PCI. This novel prognostic scale, termed SIC exhibited a good discriminatory power and goodness-of-fit in derivation and validation database, which had greater predictive accuracy than SI for in-hospital adverse events. The discriminatory capacity of SIC for in-hospital death was non-inferior to the GRACE scale, and SIC was a better predictor than TIMI risk score. In addition, SIC displayed modest discriminatory ability for identification of CI-AKI and bleeding. The cumulative 1-year mortality was significantly higher in patients with a high SIC. Use of SIC could provide prognostic information to aid in early rapid risk assessment of patients with STEMI undergoing PCI.

The mortality rate of patients with STEMI is high despite use of PCI and secondary preventive strategies. Many factors, including tachycardia, hypotension, and renal function have been shown to be independent predictors of early STEMI-related death (6). However, no single biomarker has been identified that can predict adverse events. Therefore, several risk scales have been established as prognostic indicators for STEMI.

The TIMI risk score was first developed for fibrinolytic-eligible patients with STEMI, and its accuracy and clinical applicability were validated in patients with STEMI undergoing PCI (16, 17). This score is recommended by contemporary guidelines for use in patients with STEMI, and is a simple bedside scoring tool that can effectively predict in-hospital mortality (18). However, TIMI is only a modest predictor of 1-year mortality (AUC = 0.73) (19). Current guidelines also recommend use of the GRACE scale as a prognostic indicator for patients with STEMI (6). The GRACE scale exhibits good discriminatory performance for short- and long-term outcomes (AUC ≥ 0.8) (20). In our study, GRACE score had higher predictive value than TIMI risk score, which agreed with the findings in a previous study (21). However, GRACE score is not routinely used in patients with STEMI because it uses a complex formula that requires computer-assisted calculation. Therefore, a simpler risk score with adequate predictive accuracy was needed for patients with STEMI.

SI was initially described in 1967, and is commonly used to assess the severity of shock in the clinical setting (22). SI has also been used for risk assessment of several conditions including trauma, stroke, and sepsis (23–25). In STEMI, sustained obstruction of blood supply results in myocardial necrosis, which can result in cardiac dysfunction (26). Epidemiological data showed that approximately 6% patients with STEMI also had cardiogenic shock (27). Cardiogenic shock was associated with significantly higher short- and long-term risk for mortality, as evidenced by an increase from 25 to 50% (28). Therefore, SI is considered a viable risk projection model for patients with STEMI. However, the predictive accuracy of SI is not adequate. The AUC values for in-hospital and one-year mortality were 0.703 and 0.660, respectively (10, 29). Renal dysfunction is believed to be a risk factor for patients with STEMI (1, 6). Cywinski et al. showed that estimated renal function was a better prognostic indicator than Scr (30). CCr by Cockcroft-Gault has adequate discriminatory ability, with an AUC > 0.8 for prediction of poor outcomes, which was better than other equations for glomerular filtration rate estimation in patients with acute coronary syndrome (31, 32). In addition, renal function is an important element in the GRACE, Mehran, and CRUSADE scales (7, 11, 12). Therefore, we hypothesized that addition of CCr to SI could result in better predictive accuracy in patients with STEMI undergoing PCI. Our results showed that the predictive value of SIC for in-hospital death was equivalent to that of GRACE and better than that of TIMI risk score. In addition, SIC did better in predicting MACEs than these two scales. SIC is calculated using only 3 variables, which may result in greater use by clinicians.

In the present study, we also explored the discriminatory ability of SIC for CI-AKI and bleeding. These complications occur frequently in patients with STEMI undergoing PCI, and are associated with poor prognosis (4, 5). The Mehran score consists of 8 variables and has been validated as an accurate predictor of CI-AKI (7, 33). The procedural variables (contrast media volume) included in this score have limited its early application. In our study, although SIC was similar to Mehran score for prediction of CI-AKI, it could serve as a rapid and effective tool for early prediction of CI-AKI. Given that blood pressure, heart rate, and renal function contribute to bleeding, we compared the predictive abilities of SIC and CRUSADE (12). SIC had equivalent predictive value for bleeding as CRUSADE.

SIC presented in this study has several advantages. First, it included 3 risk factors that were easily collected and calculated. Second, although it has less variables, it has similar discriminatory ability with guidelines recommending risk-stratified score. Third, it shared the same risk factors with previous scores for predicting CI-AKI and bleeding. By using these simple data (heart rate, systolic blood pressure, and CCr), SIC can be used to assess and stratify the risk for multiple events rapidly and precisely, at no additional cost or effort.

### Study Limitation

Our study suffered from several limitations. First, this study was retrospective, of moderate-scale, and from a single center, and this score should be externally validated in a large-scale multicenter study. Second, blood pressure was not invasively measured in catheterization room, which is more reliable than sphygmomanometer. However, it could represent the contemporary and real-world clinical practice. Third, the predictive value of SIC was only validated in patients with STEMI undergoing PCI, and caution should be used when using this scale to evaluate patients not undergoing PCI. Fourth, the proportion of female in this study was relatively small, the predictive ability should be validated in another female cohort of STEMI, despite the good discrimination power in the subgroup analysis.

## Conclusion

In conclusion, we showed that SI was a good prognostic indicator in patients with STEMI undergoing PCI, but its discriminatory ability was insufficient. Addition of renal function to SI resulted in better predictive power and good calibration. SIC had similar predictive value for in-hospital death as GRACE score, and better discrimination power than TIMI risk scales. In addition, SIC showed modest predictive value for CI-AKI and bleeding. Higher SIC was an independent predictor for 1-year mortality. This indicator might provide prognostic information for early and rapid risk assessment of patients with STEMI undergoing PCI.

## Data Availability Statement

The raw data supporting the conclusions of this article will be made available by the authors, without undue reservation.

## Ethics Statement

The studies involving human participants were reviewed and approved by Ethics Committee of Guangdong Provincial People's Hospital. Written informed consent for participation was not required for this study in accordance with the national legislation and the institutional requirements.

## Author Contributions

J-yC, D-qY, and GL contributed to the conception or design of the study. PR, X-bW, Y-wL, J-lH, X-yH, and J-qY contributed to the acquisition, analysis, or interpretation of data. PR, X-bW, and Y-wL drafted the manuscript. J-yC and D-qY critically revised the manuscript. All the authors gave final approval and agreed to be accountable for all aspects of work ensuring integrity and accuracy.

## Conflict of Interest

The authors declare that the research was conducted in the absence of any commercial or financial relationships that could be construed as a potential conflict of interest.

## Abbreviations

AUC: area under the curve
CCr: creatinine clearance rate
CI: confidence interval
CI-AKI: contrast-induced acute kidney injury
CRUSADE: Can Rapid Risk Stratification of Unstable Angina Patients Suppress Adverse Outcomes With Early Implementation of the American College of Cardiology/American Heart Association Guidelines
GRACE: Global Registry of Acute Coronary Events
LMCA: left main coronary artery
HR: hazard ratio
IABP: intra-aortic balloon pump
IDI: integrated discrimination improvement
LVEF: left ventricular ejection fraction
MACEs: major adverse clinical events
NRI: net reclassification improvement
PCI: percutaneous coronary intervention
ROC: receiver operating characteristic
Scr: serum creatinine
SI: shock index
SIC: shock Index-C
STEMI: ST-segment elevation myocardial infarction
TIMI: Thrombolysis in Myocardial Infarction
TVR: target vessel revascularization.
