# Supplementary material for: Shock Index-C: An Updated and Simple Risk-Stratifying Tool in ST-Segment Elevation Myocardial Infarction
Source: Front Cardiovasc Med. 2021 Jun 15;8:657817. doi: 10.3389/fcvm.2021.657817 (PMC8241092; doi:10.3389/fcvm.2021.657817)

**Supplement**

**Table S1. Development Series: Multivariable Logistic Regression Analysis for in-hospital mortality.**

| Clinical variables | OR | 95%CI | p-value |
| --- | --- | --- | --- |
| Female | 1.43 | 0.58,3.52 | 0.433 |
| Smoke | 0.43 | 0.17,1.10 | 0.079 |
| Cardiac arrest before admission | 5.45 | 1.25,23.79 | 0.024 |
| Shock index×100 | 1.02 | 1.01,1.03 | 0.005 |
| Killip≥2 | 0.90 | 0.38,2.10 | 0.804 |
| CCr | 0.97 | 0.95,0.98 | <0.001 |
| LVEF | 0.96 | 0.93,0.99 | 0.015 |
| IABP | 4.18 | 1.82,9.62 | 0.001 |
| LMCA intervention | 1.97 | 0.60,6.50 | 0.264 |

Abbreviations: CCr，creatinine clearance rate；IABP, intra-aortic balloon pump; LVEF, left ventricular ejection fraction; LMCA, left main coronary artery.

**Table S2. AUC, net reclassification improvement and integrated discrimination improvement for predicting in-hospital adverse events**

| **Model comparison** | AUC comparison | P value | NRI% (95% CI) | P value | IDI% (95% CI) | P value |
| --- | --- | --- | --- | --- | --- | --- |
| **Death** |  |  |  |  |  |  |
| SIC vs. GRACE | 0.874 vs. 0.859 | 0.453 | 16.5(-2.1,35.1) | 0.082 | 3.6(-0.3,7.6) | 0.072 |
| SIC vs. TIMI | 0.874 vs. 0.822 | 0.006 | 20.1(0.7,39.5) | 0.042 | 7.5(2.9,12.1) | 0.001 |
| **MACEs** |  |  |  |  |  |  |
| SIC vs. GRACE | 0.837 vs. 0.804 | 0.008 | 17.4(5.0,29.8) | 0.006 | 4.4(1.6,7.3) | 0.002 |
| SIC vs. TIMI | 0.837 vs. 0.762 | <0.001 | 53.6(40.7,66.4) | <0.001 | 11.1(8.2,13.9) | <0.001 |
| **CI-AKI** |  |  |  |  |  |  |
| SIC vs. Mehran | 0.707 vs. 0.749 | 0.029 | -41.5(-54.8,-28.2) | <0.001 | -4.2(-6.0,-2.4) | <0.001 |
| **Bleeding** |  |  |  |  |  |  |
| SIC vs. CRUSADE | 0.732 vs. 0.743 | 0.380 | -8.7(-20.8,3.5) | 0.161 | 0.3(-1.3,1.9) | 0.691 |

Abbreviations: AUC, area under the curve; CI-AKI, contrast-induced acute kidney injury; CRUSADE, Can Rapid Risk Stratification of Unstable Angina Patients Suppress Adverse Outcomes With Early Implementation of the American College of Cardiology/American Heart Association Guidelines; GRACE, Global Registry of Acute Coronary Events; IDI, integrated discrimination improvement; MACEs, major adverse clinical events; NRI, net reclassification improvement.

**Table S3. Multivariable analysis for one-year death**

| Variable | SIC | | | | | |
| --- | --- | --- | --- | --- | --- | --- |
|  | 2nd vs. 1st tertile | P value | 3rd vs. 1st tertile | P value | >10 vs. ≤10 | P value |
| Unadjusted HR (95%CI) | 3.64(1.35,9.80) | 0.011 | 20.10(8.20,49.25) | <0.001 | 8.16(5.63,11.81) | <0.001 |
| Adjusted HR# (95%CI) | 2.86(1.06,7.74) | 0.038 | 7.80(3.08,19.79) | <0.001 | 3.13(2.01,4.87) | <0.001 |

#Adjusted for female gender, smoke, diabetes, hypertension, weight, cardiac arrest before admission, time to admission>4 hours, Killip≥2, anemia, LVEF, IABP and LMCA intervention.

Abbreviations: CI, confidence interval; HR, hazard ratio; IABP, intra-aortic balloon pump; LMCA, left main coronary artery; LVEF, left ventricular ejection fraction.

**Figure S1.** Flow chart of the study population inclusion

**

**

**Figure S2.** Prevalence of in-hospital adverse events


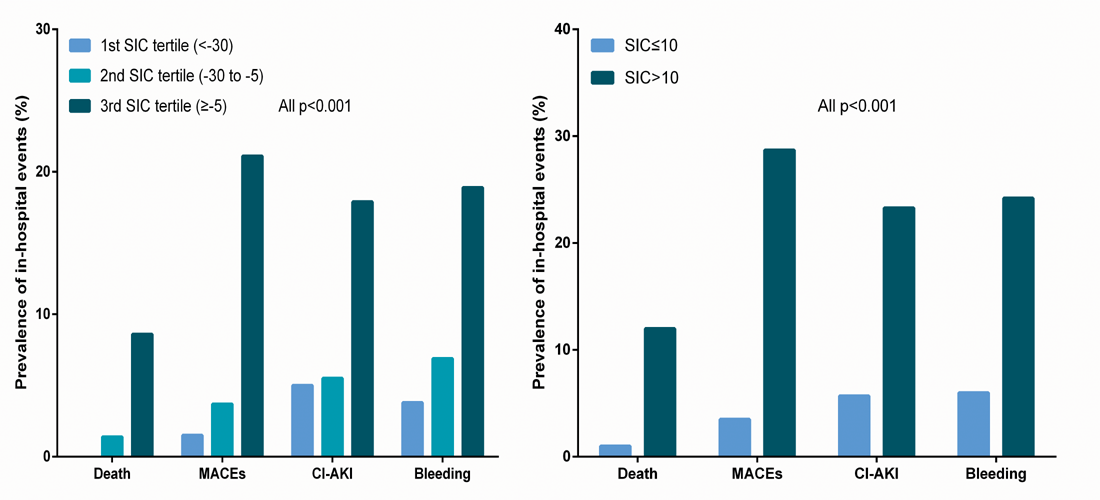


**Figure S3.** Cumulative incidence curve for one-year death.


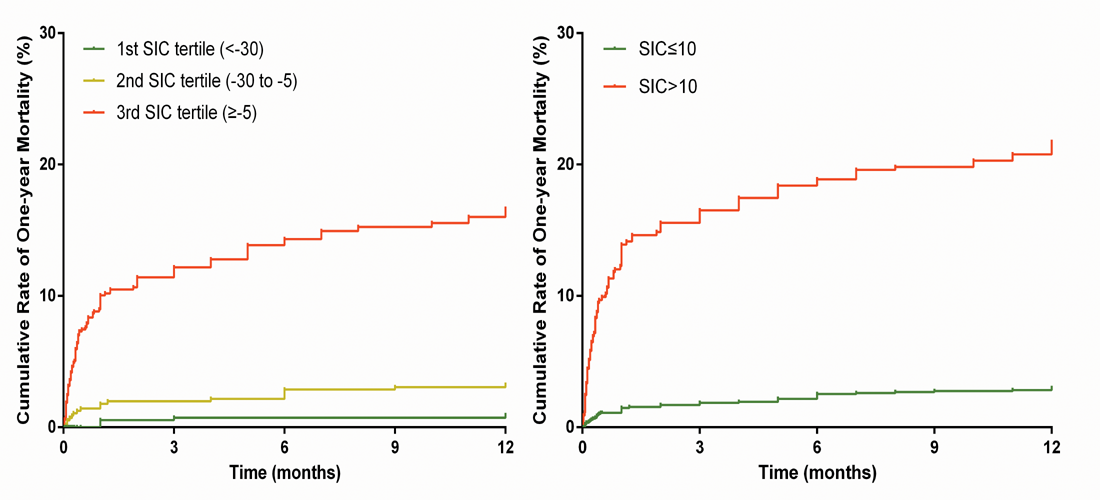

Supplement: Supplementary file 1 [file Data_Sheet_1.doc]
